# The association between laminin and microglial morphology *in vitro*

**DOI:** 10.1038/srep28580

**Published:** 2016-06-23

**Authors:** Wing Yip Tam, Ngan Pan Bennett Au, Chi Him Eddie Ma

**Affiliations:** 1 Department of Biomedical Sciences, City University of Hong Kong, Tat Chee Avenue, Hong Kong; 2 Centre for Biosystems, Neuroscience, and Nanotechnology, City University of Hong Kong, Tat Chee Avenue, Hong Kong; 3 State Key Laboratory in Marine Pollution, City University of Hong Kong, Tat Chee Avenue, Hong Kong

## Abstract

Microglia are immune cells in the central nervous system (CNS) that contribute to primary innate immune responses. The morphology of microglia is closely associated with their functional activities. The majority of microglial studies have focused on the ramified or amoeboid morphology; however, bipolar/rod-shaped microglia have recently received much attention. Bipolar/rod-shaped microglia form trains with end-to-end alignment in injured brains and retinae, which is proposed as an important mechanism in CNS repair. We previously established a cell culture model system to enrich bipolar/rod-shaped microglia simply by growing primary microglia on scratched poly-D-lysine (PDL)/laminin-coated surfaces. Here, we investigated the role of laminin in morphological changes of microglia. Bipolar/rod-shaped microglia trains were transiently formed on scratched surfaces without PDL/laminin coating, but the microglia alignment disappeared after 3 days in culture. Amoeboid microglia digested the surrounding laminin, and the gene and protein expression of laminin-cleaving genes *Adam9* and *Ctss* was up-regulated. Interestingly, lipopolysaccharide (LPS)-induced transformation from bipolar/rod-shaped into amoeboid microglia increased the expression of *Adam9* and *Ctss*, and the expression of these genes in LPS-treated amoeboid-enriched cultures remained unchanged. These results indicate a strong association between laminin and morphological transformation of microglia, shedding new light on the role of bipolar/rod-shaped microglia in CNS repair.

Microglia are the immune cells of the central nervous system (CNS) that are crucial for repair after injury. These cells release inflammatory cytokines and phagocytose damaged cells around the injury site to facilitate wound healing. It has been suggested that the morphology of microglia is highly associated with the function^1^,^2^,^3^ and microenvironment^4^,^5^ of these cells. Ramified microglia with motile processes and protrusions are regarded as surveillance cells, which actively sample the neuronal microenvironment^1^,^6^,^7^,^8^. Amoeboid microglia, with fewer or even absent processes, are highly motile and active phagocytes^2^,^3^. Compared to these two well-studied morphologies, bipolar/rod-shaped microglia are much less well characterized. However, it is increasingly evident that bipolar/rod-shaped microglia play a key role in CNS repair. Recent studies have shown that the clustering of bipolar/rod-shaped microglia aligned end-to-end at the damage site after traumatic or ischaemic brain injury^9^,^10^,^11^. We and others have also shown that these bipolar/rod-shaped microglia are highly proliferative^2^,^5^,^12^,^13^. Additionally, an increase in the number of proliferating microglia minimizes brain damage because the elimination of proliferating microglia induced more severe damage after ischaemic insult in the cortex of genetically engineered mice^14^,^15^. The local proliferation of bipolar/rod-shaped microglia, but not infiltrating peripheral macrophages, was detected in the retina after optic nerve injury^16^. In a laser-induced ocular hypertension (OHT) mouse model, the chain alignment of bipolar/rod-shaped microglia was observed only in the nerve fibre layer of the OHT retina, indicating a key role for bipolar/rod-shaped microglia in axonal degeneration^17^. These studies clearly demonstrate the critical role of proliferating bipolar/rod-shaped microglia in CNS repair.

The diverse extracellular matrix (ECM) subtypes present in the brain not only contribute to the normal physiology of this organ but are also heavily involved in CNS repair after injury^18^,^19^,^20^. CNS injury results in the deposition of the ECM into the neural interstitial matrix, associated with the alteration of microglial morphology^21^,^22^,^23^. Among ECM proteins that are differentially expressed at the lesion site, elevated laminin expression after CNS injury seems to be beneficial to CNS repair. Laminin provides a growth-permissive microenvironment that promotes the neurite outgrowth of primary cortical and hippocampal neurons *in vitro*^24^,^25^. The pronounced elevation of laminin expression was induced shortly after ischaemic^21^ (i.e., 6 hours) and traumatic brain injury^19^,^23^ (i.e., 24 hours), showing peak expression 3 days after injury^23^. In addition to a role in promoting axonal growth, laminin up-regulation after CNS injury has been correlated with the transformation of ramified microglia into amoeboid microglia. For example, a rapid morphological transformation of microglia was observed in ischaemic brain injury 6 hours after middle cerebral artery occlusion (MCAO), while laminin expression was dramatically increased^21^. The increase in the number of amoeboid microglia observed in the MCAO animal model was consistent with the fact that a majority of microglia adopted amoeboid morphology on poly-D-lysine (PDL)/laminin-coated culture dishes^4^,^5^. The vast majority of microglial studies on CNS injury models have focused on ramified or amoeboid morphologies. To our knowledge, there are currently no studies on laminin localization and bipolar/rod-shaped microglia colonization in culture or animal models.

Here, we examined the potential association between laminin and trains of bipolar/rod-shaped microglia using our previously described culture model system^5^. We showed that bipolar/rod-shaped microglia aligned only within the scratched area of the PDL/laminin-coated surface, and the alignment patterns were similar to those observed in the brain after CNS injury *in vivo*^9^,^10^,^16^. The microglia became amoeboid on the non-scratched area of the PDL/laminin-coated surface^5^. In the present study, we demonstrated that laminin was essential in maintaining the bipolar/rod-shaped microglia alignment. Stable microglia alignment could be formed and maintained only on the scratched area of a PDL/laminin-coated surface but not on the scratched area without the PDL/laminin coating. We also observed the degradation of extracellular laminin by amoeboid microglia along with the up-regulation of the mRNA expression of the laminin-cleaving genes *a disintegrin and a metalloproteinase domain 9* (*Adam9*) and *cathepsin S* (*Ctss*) in amoeboid-enriched cultures compared with bipolar/rod-enriched cultures. More importantly, the lipopolysaccharide (LPS)-induced transformation of bipolar/rod-shaped microglia into an amoeboid morphology on the scratched area of a PDL/laminin-coated surface also induced the up-regulation of *Adam9* and *Ctss* gene expression. The expression of *Adam9* and *Ctss* remained unchanged on non-scratched PDL/laminin-coated culture dishes that primarily contained amoeboid microglia after LPS treatment. Taken together, the results of the present study indicate a close association between laminin and morphological changes in microglia.

## Results

### Laminin-coated surface stabilizes trains of bipolar/rod-shaped microglia alignments *in vitro*

**Figure 1:**
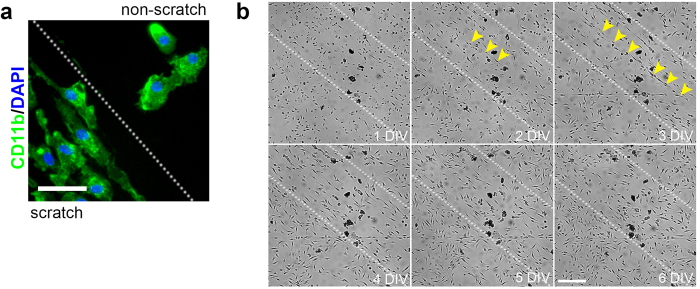
Laminin stabilizes the bipolar/rod-shaped microglia alignment. **(a)** Bipolar/rod-shaped and amoeboid microglia were colonized in the scratched (left to the line) and non-scratched areas, respectively, of a PDL/laminin-coated surface. Green: CD11b; Blue: DAPI. **(b)** Bipolar/rod-shaped microglia (yellow arrowheads) trains were unstably formed on the scratched area (area within the two dotted lines) of the culture plate without PDL/laminin-coating before 3 DIV. Scale bars: 50 μm in a; 250 μm in b.

Our previous study showed that microglia form trains of bipolar/rod-shaped microglia alignments and become stabilized on the scratched area of a PDL/laminin-coated surface after 6 days *in vitro* (DIV) (Fig. 1a)^5^. Therefore, we examined whether a physical scratch alone is sufficient to promote the alignment of bipolar/rod-shaped microglia grown on culture dishes without PDL/laminin-coating. Interestingly, we observed bipolar/rod-shaped microglia trains in the scratched area at 2 DIV (Fig. 1b, as indicated with yellow arrowheads). However, the microglia alignment was unstable and disappeared after 3 DIV when these microglia became indistinguishable from the microglia in the non-scratched area, which displayed randomized morphologies with non-directional alignment (Fig. 1b). These data showed that physical scratch alone induced the formation of bipolar/rod-shaped microglia trains, but failed to maintain a stable directional microglia alignment. In contrast, laminin might play a role in stabilizing the microglia alignment because bipolar/rod-shaped microglia trains could be maintained for at least 6 DIV^5^.

### Bipolar/rod-shaped microglia alignments form in the laminin-free scratched area

**Figure 2:**
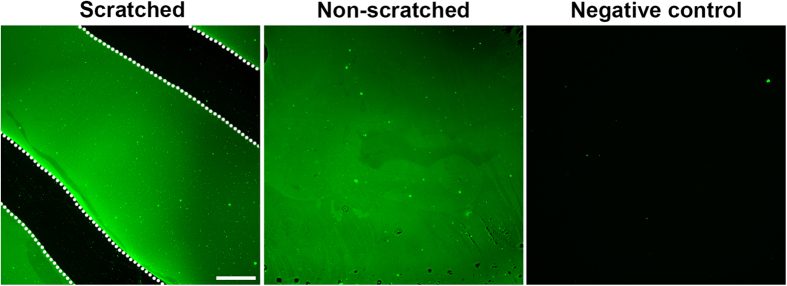
Physical scratch removes the laminin coating and generates a laminin-free area. Scratching was performed using a P200 pipette tip. Most, if not all, of the laminin coating was removed in the scratched area. The area between the two white dotted lines corresponds to the laminin-free areas generated through physical scratches (areas in black colour, left panel). Laminin was evenly distributed in the non-scratched areas (areas in green colour, middle panel). The negative control was obtained without the addition of the anti-laminin primary antibody (right panel). Scale bar: 500 μm.

Bipolar/rod-shaped microglia colonize only on the scratched area of a PDL/laminin-coated surface^5^, while physical scratches alone did not maintain stable directional bipolar/rod-shaped microglia trains (Fig. 1). We therefore hypothesized that both physical scratch and laminin coating are required to stabilize directional bipolar/rod-shaped microglia trains. We first examined the laminin distribution on the PDL/laminin-coated surfaces after scratches using an anti-laminin antibody. The scratches generated using a P200 pipette tip completely removed the laminin coating and produced a laminin-free area between non-scratched areas (Fig. 2). These data indicated that the sandwiching of microglia between laminin-coated surfaces facilitates the formation of bipolar/rod-shaped microglia alignments.

### Amoeboid microglia digest the laminin substrate and up-regulate the expression of laminin-cleaving proteins

**Figure 3:**
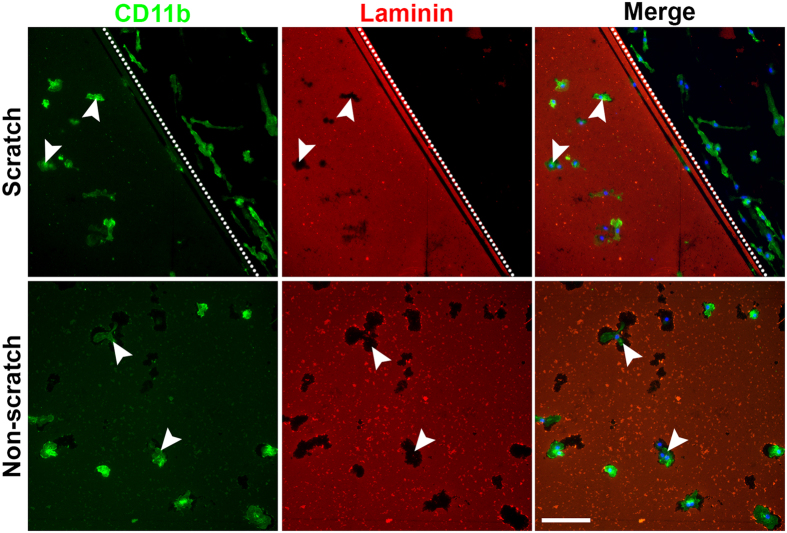
Amoeboid microglia digest the laminin coating, leaving laminin-free zones around the cells. Amoeboid microglia (white arrowheads) digested the surrounding laminin on non-scratched areas of the PDL/laminin-coated surface, leaving some laminin-free zones. The bipolar/rod-shaped microglia colonized only on the scratched area. Scale bar: 100 μm.

**Figure 4:**
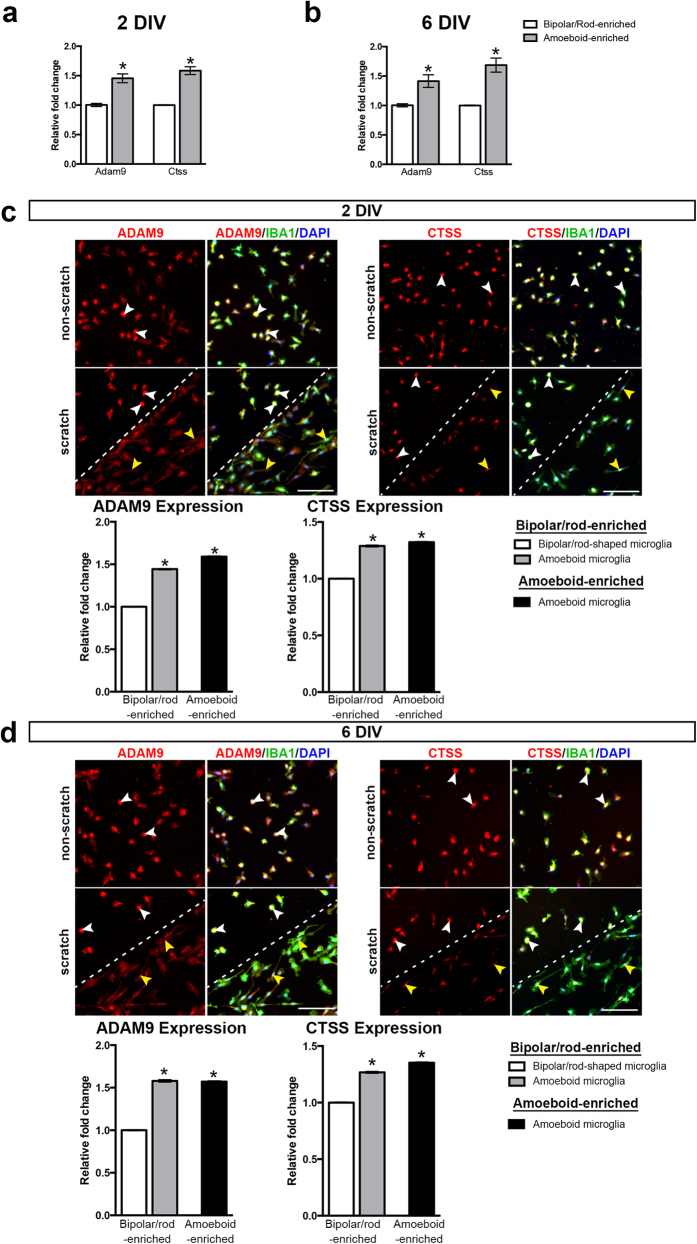
mRNA and protein expression of Adam9 and Ctss are significantly increased in amoeboid microglia. **(a)** A significant increase in the mRNA expression of *Adam9* and *Ctss* was observed in amoeboid-enriched cultures compared with bipolar/rod-enriched cultures at 2 DIV (n = 6). **(b)** At 6 DIV, the mRNA expression of *Adam9* and *Ctss* remained significantly increased in amoeboid-enriched cultures (n = 4). *P < 0.05, Student’s *t*-test. **(c)** Representative fluorescence micrographs showed strong Adam9 and Ctss immunoreactivity in amoeboid microglia (white arrowheads) compared with bipolar/rod-shaped microglia (yellow arrowheads). The quantification of the fluorescence intensity of Adam9 and Ctss confirmed the immunostaining results at 2 DIV. **(d)** Representative fluorescence micrographs showed that the protein expression of Adam9 and Ctss remained high in amoeboid microglia (white arrowheads) compared with bipolar/rod-shaped microglia (yellow arrowheads). The quantification of Adam9 and Ctss fluorescence intensity confirmed the observation at 6 DIV. Scale bar: 100 μm. We analysed approximately 600–1000 bipolar/rod-shaped or amoeboid microglia from scratched or non-scratched PDL/laminin-coated surface, respectively (n = 3; mean ± SEM; *P < 0.05, one-way ANOVA, followed by Bonferroni’s post hoc test).

We further investigated the potential role of laminin and physical scratching in regulating the morphological changes of microglia *in vitro.* Interestingly, we observed noticeable small laminin-free zones around amoeboid microglia in the non-scratched area (Fig. 3, white arrowheads). It is likely that the amoeboid microglia digested the laminin coating since amoeboid microglia phagocytose both non-oxidized and oxidized laminin^26^. To investigate the potential association between laminin-cleaving proteins and the morphology of microglia, we performed an enrichment analysis using the Gene Ontology (GO) database (amigo.geneontology.org). We identified 28 mouse genes using the search term “laminin binding” (GO: 0043236). After further literature research, we found that 5 of the 28 genes were expressed in microglia, and only Adam9 and Ctss were closely associated with laminin-cleaving activity. We therefore examined whether the degradation of laminin by amoeboid microglia is associated with the expression of two laminin-cleaving genes, *Adam9* and *Ctss*^27^,^28^, which are both expressed in microglia^29^,^30^,^31^. As expected, we observed that the mRNA levels of *Adam9* and *Ctss* were remarkably up-regulated in amoeboid microglia compared with bipolar/rod-shaped microglia at 2 DIV (Fig. 4a). At 6 DIV, both *Adam9* and *Ctss* remained significantly up-regulated in amoeboid microglia (Fig. 4b). To further validate the mRNA expression data, we examined the protein expression of ADAM9 and CTSS in bipolar/rod-shaped and amoeboid microglia. Consistent with the qPCR analysis, the protein expression of both ADAM9 and CTSS was significantly higher in amoeboid microglia than in bipolar/rod-shaped microglia at 2 DIV (Fig. 4c) and 6 DIV (Fig. 4d), respectively. These data collectively indicated that amoeboid microglia have higher laminin-cleaving activity compared with bipolar/rod-shaped microglia, and bipolar/rod-shaped microglia primarily colonized in the laminin-free scratched area.

### LPS-induced transformation of bipolar/rod-shaped microglia into amoeboid microglia increases the expression of *Adam9* and *Ctss*

**Figure 5:**
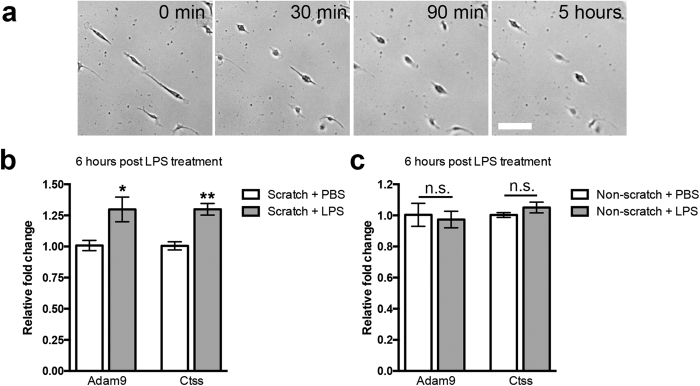
Elevated mRNA expression of *Adam9* and *Ctss* in amoeboid microglia that transformed from bipolar/rod-shape microglia after LPS treatment. (**a**) LPS-treated bipolar/rod-shaped microglia transformed into amoeboid microglia in 30 minutes and maintained this morphology for a few hours. (**b**) LPS induced an increased in mRNA expression of *Adam9* and *Ctss* in the amoeboid microglia transformed from bipolar/rod-shaped microglia at 6 hours after culture on scratched PDL/laminin-coated culture dishes (n = 4). (**c**) LPS did not induce any change in the mRNA expression of *Adam9* and *Ctss* in amoeboid microglia at 6 hours after culture on non-scratched PDL/laminin-coated culture dishes (n = 4). *P < 0.05, Student’s *t*-test.

LPS induced the activation of microglia and transformation into an amoeboid morphology in our cell culture model system^5^. The microglia were cultured on scratched (bipolar/rod-enriched cultures) or non-scratched (amoeboid-enriched cultures) PDL/laminin-coated culture dishes for 6 days. The formation of bipolar/rod-shaped microglia trains was observed only in the scratched area. At 6 DIV, LPS or PBS was added to the microglia cultures. LPS induced the rapid transformation from bipolar/rod-shaped microglia into the amoeboid morphology within 30 minutes (Fig. 5a), and the amoeboid microglia remained in the amoeboid morphology on non-scratched plates at 6 hours after LPS treatment, as shown in our previous study^5^. To further define the potential link between laminin-cleaving proteins and morphological changes in microglia, we quantified the mRNA expression of *Adam9* and *Ctss* in bipolar/rod- and amoeboid-enriched cultures after treatment with PBS or LPS using qPCR analysis. In bipolar/rod-enriched cultures, the *Adam9* and *Ctss* expression significantly increased at 6 hours after LPS treatment compared with that in the PBS controls (Fig. 5b), whereas the expression of *Adam9* and *Ctss* in LPS-treated amoeboid-enriched cultures remained unchanged (Fig. 5c). These results indicate that amoeboid microglia regained laminin cleavage activity through the up-regulation of laminin-cleaving gene expression after the transformation from the bipolar/rod-shaped morphology. More importantly, LPS did not induce the up-regulation of laminin-cleaving proteins in amoeboid-enriched cultures. Taken together, our data showed that the alteration of *Adam9* and *Ctss* expression levels are well correlated with the morphological changes of microglia.

## Discussion

Accumulating evidence suggests a crucial role for microglia in the repair and regeneration of the CNS after injury. Different morphological forms of microglia play distinctive roles during CNS damage and repair. Ramified microglia actively survey the nearby territory using motile processes^7^. Once ramified microglia detect changes in the neuronal microenvironment, such as damage or pathogen invasion, these cells are induced and transformed into amoeboid forms to perform active phagocytosis and take up the clearance roles^32^. Recently, the importance of bipolar/rod-shaped microglia in CNS repair has attracted extensive attention in glial cell research^5^,^9^,^10^,^11^,^16^. In the present study, we attempted to explore the association between laminin and microglia morphology using an *in vitro* cell culture model system. Our data suggested that in the absence of laminin, bipolar/rod-shaped microglia were still able to form trains of alignments within the first two days on the scratched surface, but these alignments rapidly disappeared. Scratching the PDL/laminin-coated surface removed most, if not all, of the laminin coating within the scratched area, where bipolar/rod-shaped microglia were colonized. Two laminin-cleaving proteins, Adam9 and Ctss, showed distinct gene and protein expression profiles in bipolar/rod- and amoeboid-enriched microglia cultures. *Adam9* and *Ctss* were up-regulated in both amoeboid-enriched microglia cultures (non-scratched PDL/laminin-coated surface), and amoeboid microglia transformed from bipolar/rod-shaped microglia after LPS treatment. An immunostaining study clearly demonstrated that amoeboid microglia exhibited strong Adam9 and Ctss immunoreactivity in bipolar/rod-shaped microglia. These studies suggested that the morphological changes of microglia are highly versatile and dynamic.

**Figure 6:**
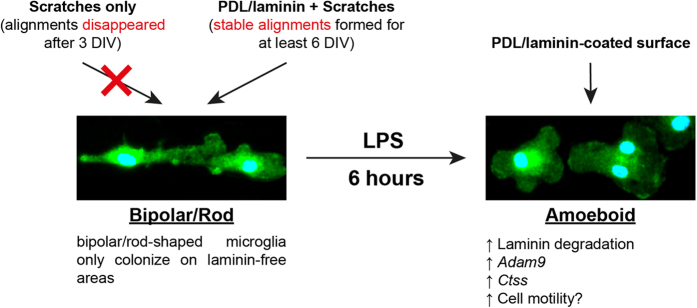
Schematic diagram illustrating the association between laminin and microglia morphology. Trains of bipolar/rod shaped microglia could not be maintained and stabilized on scratched culture dishes without a PDL/laminin coating. The scratched area of a PDL/laminin-coated surface supports stable bipolar/rod-shaped microglia trains. *Adam9* and *Ctss* up-regulation in both amoeboid-enriched and amoeboid microglia that were transformed from bipolar/rod shaped microglia is likely associated with laminin degradation and cell motility.

ECM proteins modulate microglia morphology in different cellular contexts^33^,^34^,^35^. We and others have demonstrated that laminin maintains amoeboid-shaped microglia *in vitro*^4^,^5^. In the present study, we observed that amoeboid microglia expressed higher levels of *Adam9* and *Ctss* and digested the surrounding laminin substrate. It is likely that laminin provides a substrate not only for attachment but also for consumption by these cells to sustain an amoeboid morphology. Trains of bipolar/rod-shaped microglia were transiently formed (disappeared after 3 DIV) in scratched areas without a PDL/laminin coating and subsequently, the microglia adopted randomized morphologies (Fig. 1b). We speculated that scratching might generate many tiny grooves on the surface that provide physical guidance for the formation of directional alignments. We also demonstrated that scratching the PDL/laminin-coated culture dishes removed the laminin in the scratched areas, while the laminin remained intact in the non-scratched areas (Fig. 2). These laminin and laminin-free streaks might form a laminin gradient across the surface, which could be important in maintaining the stable directional alignment of bipolar/rod-shaped microglia. In our previous study, live-cell imaging showed that microglia were attracted to the edges of the scratches on a laminin-coated surface^11^. We therefore proposed that microglia could sense differences in the laminin concentration at the boundaries between scratched and non-scratched areas to maintain bipolar/rod-shaped microglia trains (summarized in Fig. 6).

After traumatic or ischaemic brain injury, bipolar/rod-shaped microglia trains immediately form, and microglia alignments can be maintained for 1–2 weeks^9^,^10^,^16^,^17^. These bipolar/rod-shaped microglia trains have been proposed to be important in CNS repair, particularly in neuronal circuit reorganization^9^,^10^. Other studies have shown that laminin primarily increases and accumulates at the site of injury in areas with blood vessels, astrocytes and neurons during the first week after injury^19^,^21^,^23^,^36^. The up-regulation of laminin could be tightly associated with the angiogenesis, wound healing and axonal regrowth of damaged neurons after CNS injury^21^,^37^. *In vitro* studies have shown that laminin drives growth cone development, leading to the acceleration of axonal outgrowth^38^, and it enhances the regenerative capacity of CNS and PNS neurons^24^,^25^, thereby masking the inhibitory effect of CNS myelin^39^. Nevertheless, additional studies are needed to determine whether the localization of bipolar/rod-shaped microglia is directly associated with the spatiotemporal expression profiling of laminin after CNS injury. The *in vivo* CNS microenvironment would make it far too complicated and technically challenging to examine the role of individual ECM proteins in the formation of bipolar/rod-shaped microglia trains. Our culture system provides a platform for characterizing bipolar/rod-shaped microglia *in vitro*.

During the formation of bipolar/rod-shaped microglia trains, we observed the significant down-regulation of two laminin-cleaving genes, *Adam9* and *Ctss* (Fig. 4). Adam9 (MDC-9/meltrin-γ) is a member of the a disintegrin and metalloproteinase family and is a functional ligand of α6β1 integrin^40^. The secreted form of alternatively spliced Adam9 binds to α6β4 integrin, and the complex exhibits laminin-cleaving activity^27^. Ctss is a member of the lysosomal cysteine protease family, which degrades laminin^28^,^41^,^42^. In the present study, LPS induced the transformation of bipolar/rod-shaped microglia into the amoeboid morphology accompanied by the up-regulation of *Adam9* and *Ctss*. This finding indicates that the morphological changes in microglia are highly associated with the expression of laminin-cleaving proteins, given the fact that these amoeboid microglia transformed from bipolar/rod-shaped microglia, which had initially colonized in the laminin-free scratched area. We therefore proposed that bipolar/rod-shaped microglia are less capable of degrading laminin than amoeboid microglia. A recent study showed that the alignment of bipolar/rod-shaped microglia did not co-localize with other glial cells, such as oligodendrocytes, and activated astrocytes^10^, which actively produced laminin after brain injury^19^,^23^. Instead, bipolar/rod-shaped microglia trains became aligned parallel to adjacent axons^10^. We therefore hypothesized that bipolar/rod-shaped microglia align at the injury site where the laminin concentration is relatively low. Such spatiotemporal alignment of bipolar/rod-shaped microglia might be beneficial since this form of microglia could prevent the degradation of laminin through the down-regulation of *Adam9* and *Ctss*, resulting in a growth-permissive microenvironment at the injury site that would facilitate the regeneration of damaged axons. In addition, previous studies have shown that the degradation of laminin disrupts the cell-laminin interaction, leading to the apoptosis of hippocampal neurons^43^,^44^. Bipolar/rod-shaped microglia trains are formed in close proximity to axons/dendrites of injured neurons^10^. The suppression of laminin-degradation machinery in bipolar/rod-shaped microglia could maintain cell-laminin integrity, which prevents damaged neurons from undergoing apoptosis.

The up-regulation of laminin-cleaving proteins with increased laminin degradation by amoeboid microglia, as observed in the present study, might be associated with the motility and function of these cells. In general, the focal degradation of ECM proteins facilitates cell migration, as shown in different types of cells^45^,^46^. The elevated expression of Adam9 is observed in metastatic cancer cells with high motility^47^. The alternatively spliced form of Adam9 secreted from hepatic stromal cells actively degrades laminin^27^, followed by an increase in cell motility^27^,^40^. The ablation of *Ctss* impairs microglia migration, as indicated by the number of microglia that infiltrated axotomized facial motor nerves in mutant mice^29^. These findings suggest that the changes in *Adam9* and *Ctss* expression would also influence cell motility. In the present study, the elevated mRNA expression of *Adam9* and *Ctss* detected in amoeboid microglia was consistent with previous studies suggesting that microglia with this morphology are highly motile in nature and rapidly migrate towards the site of injury in response to the signals released from damaged cells after injury^48^. However, bipolar/rod-shaped microglia tend to form stable end-to-end alignments at the site of injury^9^,^10^,^16^; therefore, these cells are comparatively less motile. The differential expression of *Adam9* and *Ctss* might indicate that the cell motility in different forms of microglia is associated with the functional activities of these cells.

Taken together, the elucidation of the mechanisms underlying the regulation of the transformation into amoeboid microglia and the reversal to bipolar/rod-shaped or ramified microglia will be of great interest in designing therapeutic strategies for treating different forms of CNS injury. Further investigation of the association between the formation of bipolar/rod-shaped microglia alignment and the laminin expression levels at the injury site in animal models of CNS injury will shed new light on the roles of bipolar/rod-shaped microglia in CNS repair.

## Methods

### Animals

All experiments were conducted according to the protocols approved by the Animal Research Ethics Sub-Committee in City University of Hong Kong (Ref. A-0017) in compliance with the American Veterinary Medical Association (AVMA) Guidelines on Euthanasia that suggest exposure to carbon dioxide for animal euthanasia. C57BL/6 mice at postnatal days 1 to 3 were used for all *in vitro* experiments. The animals were provided with food and water *ad libitum*, with a 12 h light-dark cycle. We made the very best efforts to reduce the number of animals used.

### Primary microglia culture and bipolar/rod-shaped microglia enrichment

Primary microglia purification and bipolar/rod-shaped-enriched culture were performed as previously described^5^,^49^. Briefly, cerebral cortices dissected from C57BL/6 mice at postnatal days 1 to 3 were subjected to trypsinization and mechanical dissociation. For each batch of primary microglia cell preparation, we used 8 to 10 cerebral cortices for one T75 flask. Therefore, the cortices were obtained from at least 2 litters or more depending on the litter size (usually 8 pups per litter). The experiments were conducted using microglia from 2 to 3 T75 flasks. We repeated the experiments at least three times from three different batches of separately prepared microglia to obtain high reproducibility and representative data. The cell suspension was plated onto a PDL (10 μg/ml)-coated T75 flask and maintained in DMEM/10% FBS supplemented with 5 ng/ml macrophage colony-stimulating factor (M-CSF, Peprotech #315-05). After 12–14 days, the suspended and loosely attached microglial cells were collected from the culture medium after gentle shaking of the T75 flask on an orbital shaker for 15–20 minutes. The purity of the microglia obtained from the mixed primary cortical cultures was 99%, as determined by immunostaining using antibodies against a classical microglial marker, anti-IBA1 (019–19741, Wako) as previously described^5^,^49^ (Supplementary Fig. S1). The primary mixed cortical cultures were used for two rounds of microglia purification (shaking off) and maintained in culture for up to 14 days in T75 flasks. Thirty-five mm culture dishes or 8-well culture chamber slides were coated with PDL (10 μg/ml), followed by laminin (10 μg/ml, Sigma L2020). In total, 80,000 and 10,000 cells were seeded onto one 35-mm culture dish and each well of 8-well culture chamber slides, respectively. Amoeboid microglia were enriched after the primary microglia were grown on non-scratched PDL/laminin-coated culture dishes. Bipolar/rod-shaped microglia were enriched after primary microglia were seeded on scratched PDL/laminin-coated culture dishes. Multiple scratches were made on the PDL/laminin-coated culture dishes using P200 pipette tips. Purified microglia cultures were maintained for 2 to 6 days as indicated, depending on the experimental conditions.

### Immunostaining

Cultured microglia were fixed with 4% paraformaldehyde at room temperature for 15 minutes, blocked with 0.5% bovine serum albumin/0.1% Triton X-100 in PBS and stained with anti-laminin (Ab11575, Abcam), anti-CD11b (MCA74G, Serotec), anti-IBA1 (019–19741, Wako), anti-Adam9 (AF949, R&D System), and anti-Ctss (sc-271619, Santa Cruz) antibodies overnight at 4 °C. After being washed with PBS, the cultures were incubated with secondary antibodies conjugated to Alexa Fluor (Invitrogen) at room temperature for one hour. The nuclei were subsequently counterstained with DAPI.

### RNA extraction, reverse transcription and qPCR

RNA extraction, reverse transcription and qPCR analysis were performed as previously described^5^. Briefly, total RNA was extracted from the amoeboid and bipolar/rod-shaped microglia-enriched cultures using Trizol reagent (Invitrogen). Reverse transcription was performed using Superscript III First-Strand Synthesis SuperMix (Invitrogen). Quantitative real-time PCR (qPCR) was performed in triplicate using the KAPA SYBR Fast qPCR Kit (KAPA) on an ABI-7500 FAST Real-time PCR system. Relative fold changes were calculated using the 2^−ΔΔCt^ formula. *Gapdh* was used an endogenous control for normalization. The qPCR primer sequences are listed in Table 1.

### LPS treatment

LPS (Sigma L4516) was added to the amoeboid-enriched or bipolar/rod-enriched microglia cultures (final concentration of 10 μg/ml) at 6 DIV and subsequently incubated for 6 hours^5^. PBS was used as a control. Total RNA was extracted from the microglia for qPCR analysis.

### Fluorescence intensity quantification

Sixteen non-overlapping images of microglia immunostained with anti-ADAM9 or anti-CTSS antibodies were obtained using a Nikon Eclipse Ni-E microscope equipped with a motorized stage and a 20 × objective. The contours of the microglia (i.e., IBA1^+ve^) were manually outlined and selected as the region of interest (ROI) using an ImageJ plugin. The fluorescence intensity of Adam9 and Ctss within each ROI representing a single microglia was quantified using ImageJ. The microglia from each condition were also immunostained simultaneously, and the samples were imaged with identical settings under the same magnification, gain level and exposure time. Microglia images were obtained and quantified from at least three separate experiments. We analysed approximately 600–1000 bipolar/rod-shaped or amoeboid microglia from scratched or non-scratched PDL/laminin-coating surfaces, respectively, and a representative image was selected.

### Statistical analyses

GraphPad Prism 5.0 software was used for the statistical analyses. All data are presented as the means ± SEM. Student’s *t*-test (2 groups) or one-way ANOVA with post-hoc Bonferroni analysis (more than 2 groups) were used for comparisons where appropriate, with *P *< 0.05 considered statistically significant.

## Additional Information

**How to cite this article**: Tam, W. Y. *et al*. The association between laminin and microglial morphology *in vitro*. *Sci. Rep.* **6**, 28580; doi: 10.1038/srep28580 (2016).

## Supplementary Material

Supplementary Information

## Figures and Tables

**Table 1:** qPCR primers used in this study.

Gene	Primer sequence (5′ to 3′)
Gapdh	Forward: CATGGCCTTCCGTGTTCCTA
	Reverse: CCTGCTTCACCACCTTCTTGAT
Adam9	Forward: GTCATCCAGGCCCAAGGAAA
	Reverse: TGGTCAGAGAGTAGGGAGCC
Ctss	Forward: GGAGTGAGCACCACACTTCA
	Reverse: TCCCAATGGTAGTCCAGGGT
