# Supplementary material for: The association between laminin and microglial morphology in vitro
Source: Sci Rep. 2016 Jun 23;6:28580. doi: 10.1038/srep28580 (PMC4917827; doi:10.1038/srep28580)
Supplement: Supplementary Information [file srep28580-s1.pdf]

Supplementary Information

The association between laminin and microglial morphology *in vitro*

Wing Yip Tam[1†], Ngan Pan Bennett Au[1†], Chi Him Eddie Ma[1, 2, 3]

[1]Department of Biomedical Sciences, [2]Centre for Biosystems, Neuroscience, and Nanotechnology, [3]State Key Laboratory in Marine Pollution, City University of Hong Kong, Tat Chee Avenue, Hong Kong

[†] These authors contributed equally to this work.

Correspondence to: Dr. Chi Him Eddie Ma

[1]Department of Biomedical Sciences, [2]Centre for Biosystems, Neuroscience, and Nanotechnology, [3]State Key Laboratory in Marine Pollution, City University of Hong Kong, Tat Chee Avenue, Hong Kong

Email: eddiema@cityu.edu.hk

Phone: (852)-3442-9328 Fax: (852)-3442-0549

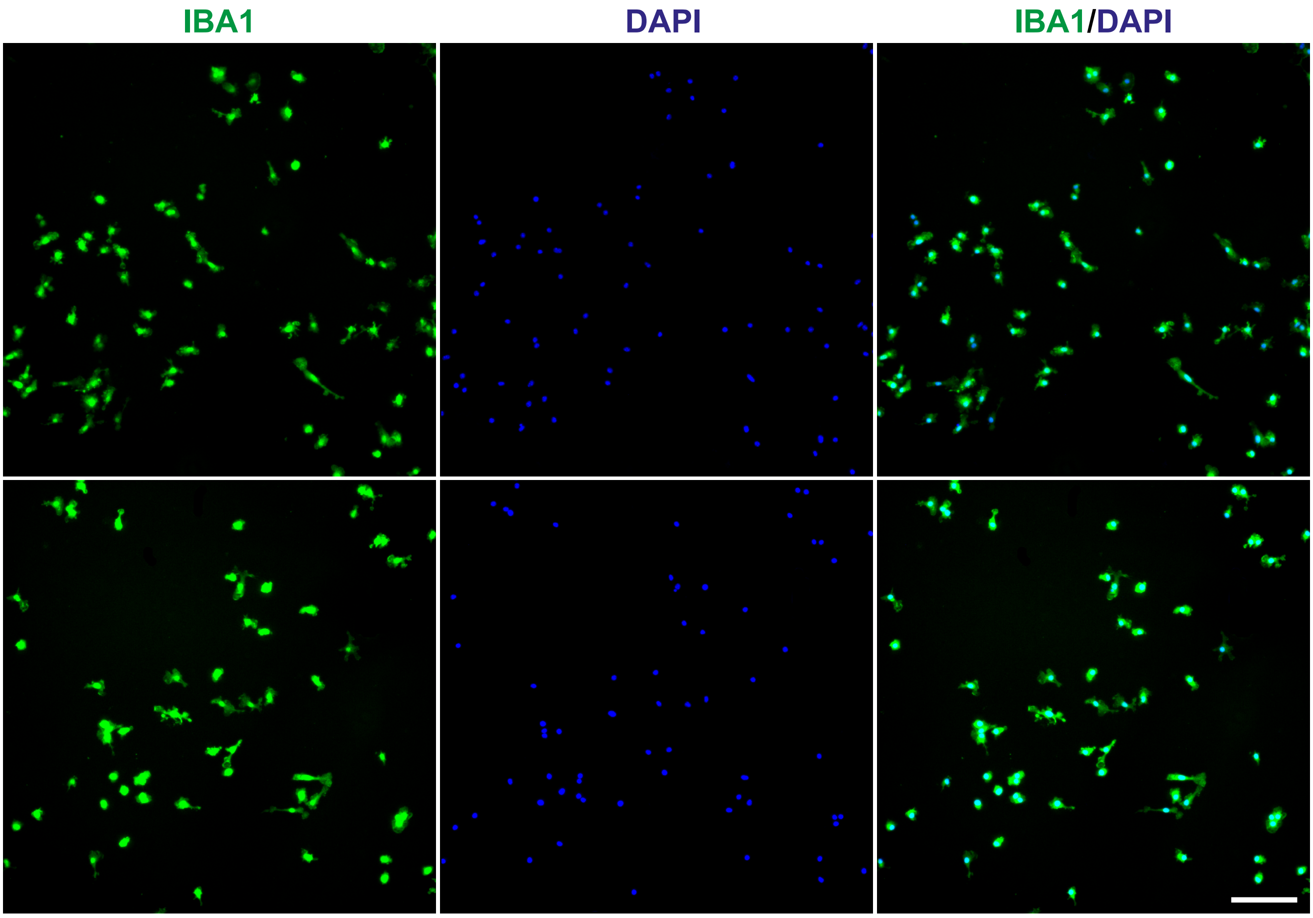

Supplementary Figure S1 | Microglia culture is highly pure. Representative fluorescence micrographs indicated that pure microglia culture could be obtained from the mixed primary cortical cultures, and the purity was 99% as determined by immunostaining using antibody against a classical microglial marker anti-IBA1 (as shown in green). DAPI was used for counterstaining of nuclei. Scale bar: 100 μm.
